# MicroRNAs: Novel Regulators Involved in the Pathogenesis of Psoriasis?

**DOI:** 10.1371/journal.pone.0000610

**Published:** 2007-07-11

**Authors:** Enikö Sonkoly, Tianling Wei, Peter C.J. Janson, Annika Sääf, Lena Lundeberg, Maria Tengvall-Linder, Gunnar Norstedt, Harri Alenius, Bernhard Homey, Annika Scheynius, Mona Ståhle, Andor Pivarcsi

**Affiliations:** 1 Dermatology and Venereology Unit, Department of Medicine, Karolinska Institutet, Stockholm, Sweden; 2 Clinical Allergy Research Unit, Department of Medicine, Karolinska Institutet, Stockholm, Sweden; 3 Department of Molecular Medicine and Surgery, Center for Molecular Medicine, Karolinska Institutet, Stockholm, Sweden; 4 Unit of Excellence in Immunotoxicology, Finnish Institute of Occupational Health, Helsinki, Finland; 5 Department of Dermatology, Heinrich-Heine University, Düsseldorf, Germany; Centre de Recherche Public-Santé, Luxembourg

## Abstract

MicroRNAs are a recently discovered class of posttranscriptional regulators of gene expression with critical functions in health and disease. Psoriasis is the most prevalent chronic inflammatory skin disease in adults, with a substantial negative impact on the patients' quality of life. Here we show for the first time that psoriasis-affected skin has a specific microRNA expression profile when compared with healthy human skin or with another chronic inflammatory skin disease, atopic eczema. Among the psoriasis-specific microRNAs, we identified leukocyte-derived microRNAs and one keratinocyte-derived microRNA, miR-203. In a panel of 21 different human organs and tissues, miR-203 showed a highly skin-specific expression profile. Among the cellular constituents of the skin, it was exclusively expressed by keratinocytes. The up-regulation of miR-203 in psoriatic plaques was concurrent with the down-regulation of an evolutionary conserved target of miR-203, suppressor of cytokine signaling 3 (SOCS-3), which is involved in inflammatory responses and keratinocyte functions. Our results suggest that microRNA deregulation is involved in the pathogenesis of psoriasis and contributes to the dysfunction of the cross talk between resident and infiltrating cells. Taken together, a new layer of regulatory mechanisms is involved in the pathogenesis of chronic inflammatory skin diseases.

## Introduction

MicroRNAs (miRNAs) are ∼22 nt noncoding RNAs that can suppress the expression of protein-coding genes by targeting cognate messenger RNAs for translational repression or, less frequently, degradation [1], [2]. In the human genome, miRNAs comprise 1-5% of all genes making them the most abundant class of regulators. The high sequence conservation of many miRNAs among distantly related organisms suggests strong evolutionary pressure and participation in essential processes [1]. Indeed, miRNAs have regulatory roles in development, differentiation, organogenesis, stem cell and germline proliferation, growth control and apoptosis. Moreover, deregulation of miRNA expression may contribute to human diseases; in particular, miRNAs are often aberrantly expressed or mutated in cancer [3]. Thus, miRNAs represent important targets for potential therapeutic and diagnostic agents [4].

While miRNAs are known to regulate cell growth, apoptosis, differentiation and morphological development, neither their expression nor roles have been characterized in skin diseases. Psoriasis is the most prevalent chronic inflammatory skin disease in adults affecting 1-3% of the population worldwide with a substantial negative impact on the patients' quality of life [5]. A complex interplay of genetic and environmental factors together with immunoregulatory abnormalities is thought to play a critical role in the pathogenesis of this disease. Keratinocytes and infiltrating immune cells play a cooperative role in the formation of psoriasis lesions; however, the exact molecular mechanisms regulating the complex interactions among resident skin cells and infiltrating immune cells are still not completely understood. Investigations analyzing the molecular background of psoriasis have identified hundreds of disease-associated genes and proteins with aberrant expression [6]–[8], however, our understanding about the regulatory networks underlying the altered expression of these genes is far from being complete.

Here, we demonstrate that psoriasis is characterized by a specific miRNA expression profile that differs from that of healthy skin or another chronic inflammatory disease, atopic eczema. Among the miRNAs overexpressed in psoriasis, we identified a keratinocyte-specific miRNA (miR-203) and a leukocyte-derived miRNA (miR-146a). The up-regulation of miR-203 in psoriatic plaques was concurrent with the down-regulation of an evolutionary conserved target of miR-203, suppressor of cytokine signaling 3 (SOCS-3), which is involved in inflammatory responses and keratinocyte functions. These results suggest that miRNAs contribute to psoriasis pathogenesis by modulating protein expression and cellular functions in both keratinocytes and infiltrating immune cells. Thus, our findings reveal a new layer of regulatory mechanisms in the pathogenesis of chronic inflammatory skin diseases.

## Results and discussion

### A characteristic miRNA signature identified in psoriasis skin

**Figure 1:**
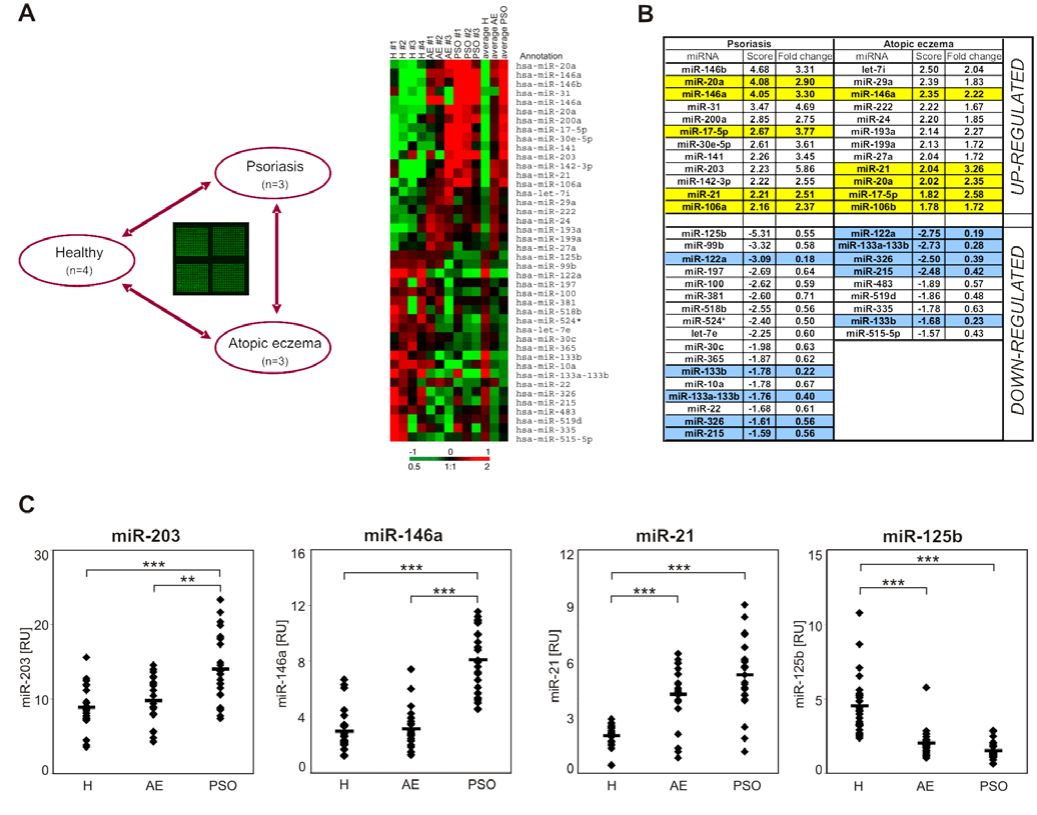
MicroRNA expression profiling in psoriasis, atopic eczema and healthy skin. (A) microRNA (miRNA) array comparison of skin from healthy individuals (H, n = 4) and lesional skin from patients with psoriasis (PSO, n = 3) and atopic eczema (AE, n = 3). Total RNA from skin biopsies was labeled and hybridized to microarrays containing probes corresponding to known miRNA sequences. The heat map summarizes the biological replicates for the skin specimens, and four technical replicates for each set. Color intensity is scaled within each row so that the highest expression value corresponds to bright red and the lowest to bright green. Gene names are listed to the right. (B) miRNAs showing more than 1.7-fold change between psoriasis and healthy skin (left) and atopic eczema and healthy skin (right) according to the SAM algorithm. miRNAs that are over-expressed in both psoriasis and in atopic eczema are highlighted in yellow, miRNAs that are down-regulated in both diseases are highlighted in blue. (C) The expressions of the functionally active, mature forms of four miRNAs were analyzed using quantitative real-time PCR in the skin of 26 healthy individuals, and lesional skin samples of 20 patients with atopic eczema and 25 patients with psoriasis. The results for individual patients and mean are shown. Data are expressed in relative units compared to U48 RNA. ***p<0.001, **p<0.01.

At present, the expression and function of miRNAs in human skin is largely unknown. To determine whether miRNAs are involved in the pathogenesis of psoriasis, we performed a comprehensive analysis of all human miRNAs registered in mirBase 8.0 (342 known human miRNAs) in skin lesions of patients with psoriasis (n = 3) and compared it to healthy human skin (n = 4) or to lesional skin from patients with a nonpsoriatic chronic inflammatory skin disease, atopic eczema (n = 3). Analysis of the microarray data showed that miRNAs are expressed in a non-random manner in psoriasis, healthy, or atopic eczema skin (Fig. 1A). Using the Significance Analysis of Microarrays (SAM) algorithm [9], we identified 29 genes that were consistently differentially expressed between psoriasis and healthy skin (Fig. 1B). Among the genes identified in the SAM analysis there were (I) miRNAs specifically up-regulated in psoriasis (e.g. miR-203), (II) miRNAs with increased expression in both psoriasis and atopic eczema (e.g. miR-21), (III) miRNAs specifically down-regulated in psoriasis (e.g. miR-99b), and (IV) miRNAs uniformly down-regulated in both skin diseases (e.g. miR-122a), as compared to healthy skin.

To confirm the results obtained by microarray profiling, we performed quantitative real-time PCR analysis of miR-203, miR-146a, miR-21 and miR-125b expression on RNA samples obtained from lesional skin of patients with psoriasis (n = 25), healthy skin (n = 26) or atopic eczema lesions (n = 20). For this, we used primers designed to amplify specifically the mature, biologically active form of these miRNAs (Fig. 1C). In accordance with the microarray data, quantitative real-time PCR results showed significantly (p<0.001) increased miR-203 levels in psoriasis skin when compared with healthy skin. Moreover, miR-203 was expressed at a significantly (p<0.01) higher level in psoriasis than in atopic eczema skin specimen. No significant up-regulation of miR-203 was observed in atopic eczema skin lesions compared with healthy skin (Fig. 1C). Similarly, miR-146a was significantly over-expressed in psoriatic lesional skin (p<0.001) but not in atopic eczema lesions when compared with healthy skin (Fig. 1C). Furthermore, miR-146a was expressed at a significantly (p<0.001) higher level in psoriasis than in atopic eczema skin samples. The psoriasis-specific overexpression of miR-203 and miR-146 suggests that they may play specific roles in the pathogenesis of psoriasis and not only a general role in skin inflammation. In contrast to miR-203 and miR-146a, miR-21 was significantly up-regulated both in psoriasis (p<0.001) and atopic eczema (p<0.001) as compared with healthy skin. miR-125b showed the opposite expression pattern to miR-21: the level of this miRNA significantly (p<0.001 for both) decreased both in psoriasis and atopic eczema. Taken together, psoriasis is characterized by a distinct miRNA expression profile in comparison with healthy skin or with atopic eczema.

### miR-203, miR-146a, miR-21 and miR-125b show distinct expression patterns in human organs and cells types

**Figure 2:**
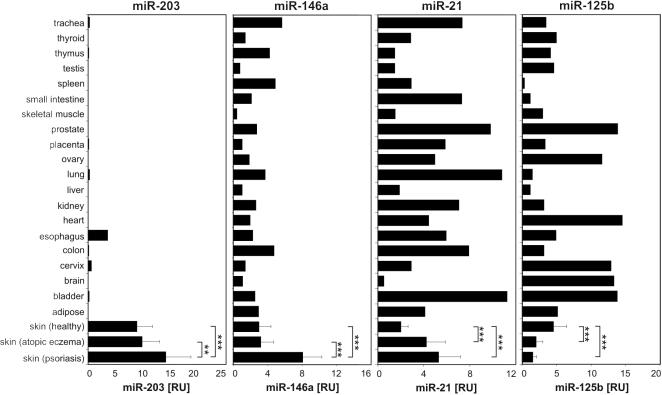
Expression of miR-203, miR-146a, miR-21 and miR-125b in human organs. The expressions of the functionally active, mature forms of miR-203, miR-21, miR-146a and miR-125b were analyzed using quantitative real-time PCR in 20 healthy organs and tissues (each a pool of three donors) as well as in healthy skin (n = 26), lesional atopic eczema (n = 20) and psoriasis (n = 25) skin samples. Error bars represent the standard error of the mean. Data are expressed in relative units compared to U48 RNA. ***p<0.001, **p<0.01.

At present, the expression pattern of the miRNAs we identified in skin is largely unknown in different organs and tissues. To obtain further insights into the function of the psoriasis-associated miRNAs, we systematically analyzed the expression of miR-203, miR-146a, miR-21 and miR-125b in skin and a panel of 20 additional human organs obtained from healthy individuals (Fig. 2). Quantitative real-time PCR analysis showed that miR-203, a miRNA specifically up-regulated in psoriasis, was expressed more than 100-fold higher in skin compared with most other organs. In addition to skin, miR-203 was only expressed at lower levels in organs that also contain squamous epithelium, esophagus and cervix. These findings suggest a specific function for this miRNA in the formation or function of squamous epithelia. In contrast to miR-203, the mature forms of miR-146a, miR-21 and miR-125b were detected in all studied organs, however, their expression showed distinct patterns. MiR-146a was highly expressed in organs containing significant number of leukocytes such as the thymus and the spleen, and showed low expression in healthy skin, suggesting that infiltrating cells express miR-146a in the skin (Fig. 2). MiR-21 showed highest expression in the lung, trachea, colon, prostate and bladder (Fig. 2), while miR-125b, a miRNA downregulated in both psoriasis and atopic eczema, was expressed mostly in organs that contain cells of ectodermal origin, including cervix, brain and bladder.

**Figure 3:**
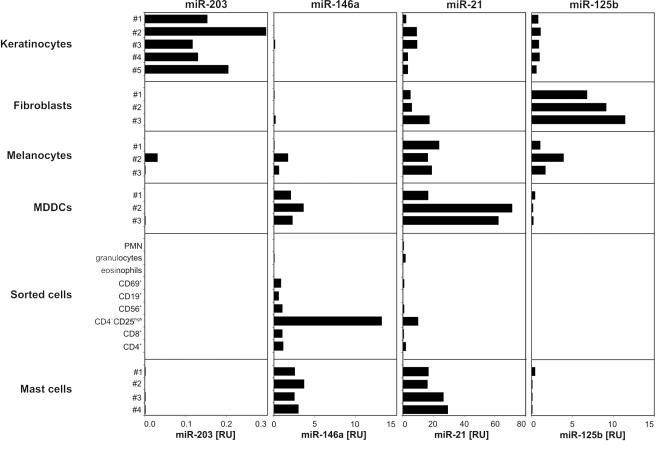
Expression of miR-203, miR-146a, miR-21 and miR-125b in the cellular constituents of the skin. The expressions of the functionally active, mature forms of miR-203, miR-146a, miR-21 and miR-125b were analyzed in the cellular constituents of the skin including primary adult keratinocytes, dermal fibroblasts, melanocytes, monocyte-derived dendritic cells (MDDCs), polymorphonuclear leukocytes (PMN), granulocytes, eosinophils, CD69^+^ cells, CD19^+^ cells, CD56^+ ^cells, CD4^+^CD25^high^ cells, CD8^+^ cells, CD4^+^ cells and mast cells using quantitative real-time PCR. Data are expressed in relative units compared to U48 RNA.

In psoriasis, there is evidence for the pathogenic relevance of several different cell types that normally occur in skin: keratinocytes [12], fibroblasts [13], monocyte-derived immunocytes [11], [14], T cells [15], and mast cells [16]. Therefore it is likely that this disease is the outcome of aberrantly activated mechanisms that do not necessarily depend on one single cell type but involve a variety of different cell populations [11], [17]. To identify the cell types expressing the identified psoriasis-associated miRNAs in the skin, we systematically analyzed the expression of miR-203, miR-146a, miR-21 and miR-125b in a panel of cells present in healthy and/or inflamed skin including both resident cells (keratinocytes, dermal fibroblasts and melanocytes) and leukocyte/immune cell subsets (CD4^+^, CD8^+^ and CD4^+^CD25^high^ T cell subsets, NK cells, granulocytes, B cells, dendritic cells and mast cells). In accordance with their expression profiles in different organs, the identified miRNAs showed a distinctive expression pattern in the studied cell types. miR-203, which was specifically expressed in skin among 21 different human organs, showed a keratinocyte-specific expression being virtually absent in all other cell types analyzed (Fig. 3). This observation suggests a role for this miRNA in keratinocyte functions in healthy skin as well as in psoriasis. By contrast, miR-146a was absent from keratinocytes and dermal fibroblasts, and it was preferentially expressed by immune cells (Fig. 3), in accordance with its high expression in immune organs (Fig. 2) and in the psoriatic inflamed skin. In particular, CD4^+^CD25^high^ regulatory T cells, monocyte-derived dendritic cells (MDDCs) and mast cells expressed miR-146a at a high level. The abundant expression of miR-146a in CD4^+^CD25^high^ cells suggests that this miRNA might influence the function of regulatory T cells in psoriatic skin. miR-21, a microRNA upregulated in both psoriasis and atopic eczema, was expressed both by structural and inflammatory cells (Fig. 2). The expression pattern of miR-125b was complementary to that of mir-146a: it was expressed at a very low level in inflammatory cells in comparison to structural cells: fibroblasts, keratinocytes and melanocytes (Fig. 2).

It is widely accepted that psoriasis is not a disease caused by one cell type but a consequence of impaired cross talk between the immune system and the structural cells of the skin [11]. Investigating the cellular distribution of miRNAs deregulated in psoriasis we found that these master switches of gene expression are expressed in cells with key roles in the pathogenesis of psoriasis. Deregulation of both keratinocyte- and leukocyte-specific miRNAs in psoriasis indicates that altered miRNA-mediated gene regulation may contribute to the disturbed cross talk between keratinocytes and immune cells. One of the most important mediators in leukocyte-keratinocyte interactions in psoriasis is tumor necrosis factor alpha (TNF-α) as evidenced by the effectiveness of TNF-α inhibitors in the treatment of psoriasis [11]. Interestingly, a recent study showed that miR-146a, one of the psoriasis-specific miRNAs, inhibits the expression of IRAK-1 and TRAF-6 proteins both of which are regulators of the TNF-α signaling pathway [10]. Hence, it is conceivable that miR-146a is involved in the pathogenesis of psoriasis via the modulation of TNF-α signaling in the skin. In contrast to miR-146, nothing is known about the function of the keratinocyte-specific miRNA, miR-203.

### A plausible link between miR-203 and keratinocyte dysfunction in psoriasis through the regulation of SOCS-3 signaling

Epidermal keratinocytes are active participants in the formation of psoriasis plaques [11]. Psoriatic keratinocytes show abnormal differentiation and proliferation, have aberrant cell signaling and produce mediators that contribute to the recruitment and activation of immune cells [11]. The specific expression of miR-203 in skin and in keratinocytes (Fig. 2 and 3) as well as its specific up-regulation in psoriasis (Fig. 1) suggested that this miRNA plays a role in the regulation of keratinocyte functions. Thus, we next focused on characterizing miR-203 in more detail.

Since miRNAs exert their effect by regulating the expression of protein-coding genes, their function can be interpreted as the sum of the function of the genes they regulate [2]. To understand the functions of miR-203 we used a two-step sequential approach: (I) using algorithms based on a systematic analysis of the structural requirements for target site function *in vivo,* we predicted genes that can be regulated by this miRNA (Table S1); (II) we investigated the biological functions of the predicted target genes. To explore whether the presence of miR-203 binding sites in the 3′ untranslated region (UTR) of mRNAs correlates with gene function, we determined if putative miR-203 targets contain significantly more or fewer genes from any given biological process than expected given the gene ontology (GO) category's frequency in the 3′UTR database (Figure S1). Out of the several thousand GO categories, the top significant (p<0.01) target categories were dominated by processes related to signal transduction, cell cycle, morphogenesis and cell growth suggesting a role for miR-203 in the regulation of these biological processes in the skin (Figure S1). Of note, these GO categories show significant overlap with the biological processes that are strongly perturbed in the psoriatic skin lesions [8].

**Figure 4:**
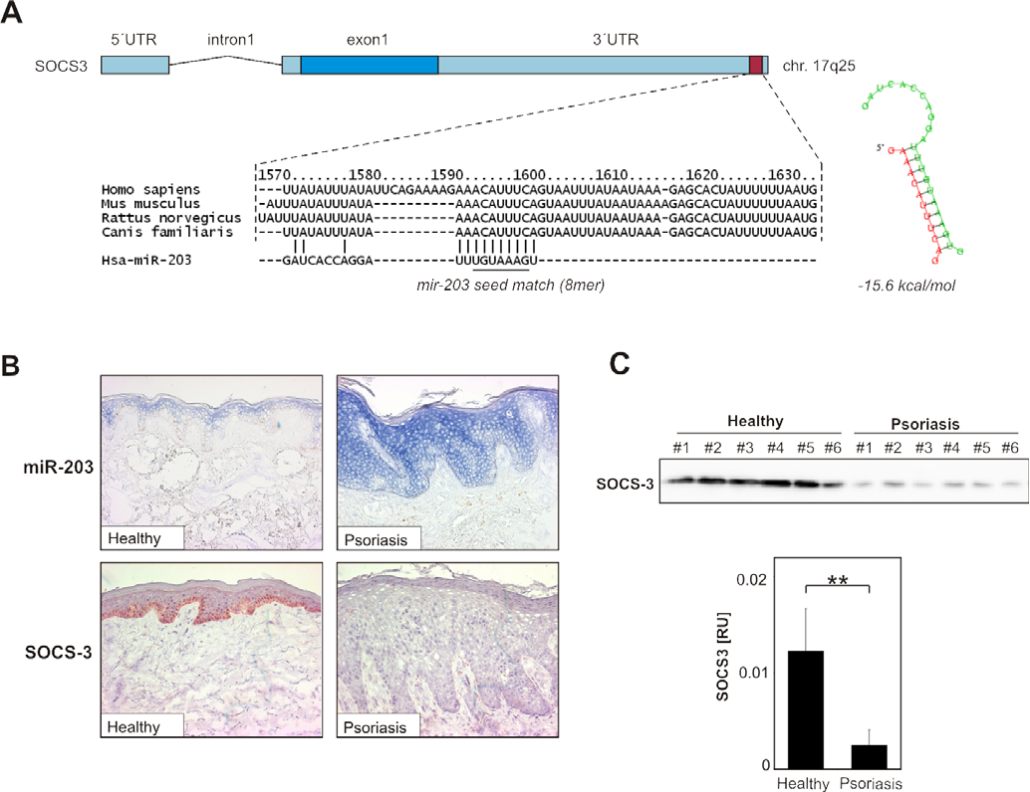
SOCS-3 may be a molecular target of miR-203 posttranscriptional repression. (A) SOCS-3 is an evolutionarily conserved target of miR-203. The putative targets site of miR-203 is highly conserved among species. The 8mer seed sequence in the 3′UTR of SOCS-3 gene corresponding to miR-203 binding site is underlined. (B) *In situ* hybridization with LNA-oligonucleotide probes specific to miR-203 in skin sections from healthy skin and psoriatic lesional skin. Data are representative of 6 healthy and 6 psoriatic individuals. Note strong staining in the suprabasal layers of the epidermis in psoriatic skin. The expression of SOCS-3 in healthy and psoriasis skin samples was detected using immunohistochemistry. Original magnification 200x. (C) The expression of SOCS-3 in skin from healthy individuals or lesional skin of psoriatic patients was analyzed using Western blot analysis. SOCS-3 protein levels are expressed as relative units. Bars represent means of SOCS-3 protein levels in healthy skin (n = 6) and psoriatic lesional skin (n = 11) ± SEM. ** p<0.01.

Among the target genes of miR-203 we focused on suppressor of cytokine signaling-3 (SOCS-3), an evolutionarily conserved high-score target of miR-203 with a 10-nucleotide complementarity to the mature, biologically active form of miR-203 in human, mouse, rat and dog (Table S1, Fig. 4A). SOCS-3 is part of a negative feedback loop in cytokine signaling inhibiting the activation of STAT3, a transcription factor whose activation in keratinocytes is essential for the development of psoriatic plaques [11], [12]. However, SOCS-3 has not been associated with psoriasis.

Next, we analyzed the expression pattern of miR-203 and SOCS-3 in lesional skin of psoriasis patients and in healthy skin (Fig. 4B). *In situ* hybridization using LNA-modified nucleotide probes revealed that miR-203 is expressed in the suprabasal layers of the epidermis in normal skin, confirming its keratinocyte-specific expression observed *in vitro* (Fig. 4B). In comparison to healthy skin, the expression of miR-203 was increased in psoriatic lesional skin in all epidermal layers (Fig. 4B), consistent with the real-time PCR results. Analysis of SOCS-3 protein expression by immunohistochemistry showed a complementary pattern with the miR-203 expression (Fig. 4B). SOCS-3 was strongly expressed by the basal layer of keratinocytes in healthy skin, while it was suppressed in the epidermis of psoriasis lesions. Down-regulation of SOCS-3 expression in psoriatic lesional skin was further confirmed by Western blot analysis, demonstrating a significant (p<0.01) decrease in SOCS-3 protein levels in psoriatic plaques as compared to healthy skin (Fig. 4C). Since the decrease of SOCS-3 protein in psoriatic skin could be due to decreased transcriptional activity of the gene, we analyzed SOCS-3 mRNA levels in psoriatic and healthy skin. However, quantitative real time PCR analysis showed no significant difference in SOCS-3 mRNA expression between psoriatic and healthy skin (data not shown), suggesting that the down-regulation of SOCS-3 in psoriasis occurs at the posttranscriptional level.

SOCS-3 deficiency leads to sustained activation of STAT3 in response to IL-6 [18], a cytokine present in the psoriasis lesions [11]. This suggests that the suppression of SOCS-3 by miR-203 in psoriatic lesions would in turn lead to constant activation of STAT3. Indeed, the psoriatic hyperplastic epidermis shows increased STAT3 activation and constitutively active STAT3 in keratinocytes leads to the spontaneous development of psoriasis in transgenic mice [12]. Thus, the up-regulation of miR-203 may have important implications for psoriasis pathogenesis by preventing the up-regulation of SOCS-3 in response to cytokines. It is intriguing to speculate that suppression of SOCS-3 in psoriatic keratinocytes leads to sustained activation of the STAT3 pathway, leading to the infiltration of leukocytes and the development of psoriatic plaques.

In addition to the modulation of inflammatory responses, SOCS-3 has also been implicated in the regulation of keratinocyte proliferation and differentiation. It has been shown that overexpression of SOCS-3 in keratinocytes leads to final differentiation and inhibits serum-stimulated proliferation [19]. MiRNA-mediated suppression of SOCS-3 expression in keratinocytes may therefore not only modulate cytokine signaling but also contribute to keratinocyte hyperproliferation and alteration in keratinocyte differentiation in psoriatic plaques.

Although here we exemplified SOCS-3 as a target of miR-203 and showed its down-regulated expression in psoriasis, it is not likely that miR-203 functions in psoriasis are mediated solely through the suppression of this protein. Instead, the function of miR-203 can be interpreted as a function of the sum of all of its target proteins and the consequence of their interactions. Thus, future research in the forthcoming years will be needed to understand fully the consequence of the deregulation of miR-203 and other miRNAs in psoriasis.

Taken together, miRNA expression patterns distinguish psoriasis from healthy skin and from another chronic inflammatory skin disease, atopic eczema. Results reported here reveal a new layer of regulatory mechanisms in the pathogenesis of chronic inflammatory skin diseases. Our data suggest that miR-203 plays a specific role in the pathogenesis of psoriasis by regulating inflammation-, proliferation- and morphogenesis-associated processes in the skin. Interestingly, miRNAs have been recently implicated in the morphogenesis of murine skin [20]. Since miRNAs are master switches that ultimately affect complex cellular processes and functions through the regulation of several proteins, miRNA-based therapies may be more effective than drugs targeting single proteins. The disease-specific miRNAs identified in our study represent potential therapeutic targets in the treatment of chronic skin inflammation.

## Materials and methods

### Patients

Both patients and healthy controls were of Caucasian origin, between 18–65 years old. Patients had not received systemic immunosuppressive treatment or PUVA/solarium/UV, for at least 1 month, and topical therapy for at least 2 weeks before skin biopsy. Four-millimeter punch biopsies were taken and snap-frozen, after written informed consent, from lesional skin of patients with moderate or severe chronic plaque psoriasis (n = 25), lesional skin of patients with moderate to severe chronic atopic eczema (n = 20), and from non-inflamed, non-irritated skin of healthy individuals (n = 26). The study was approved by the Stockholm Regional Ethicś Committee, and conducted according to the Declaration of Helsinki's principles.

### Cells

Primary human keratinocytes, dermal fibroblasts and melanocytes were isolated from healthy skin using standard protocols and cultured as described [21], [22]. Monocytes were isolated from PBMCs from healthy blood donors (Karolinska University Hospital Blood Bank, Stockholm, Sweden) using MACS separation. Immature monocyte-derived dendritic cells (MDDCs) were generated by culturing separated monocytes in the presence of GM-CSF (550 IU/ml), and IL-4 (800 IU/ml) (Biosource International, Camarillo, CA, USA) for 6 days. CD4^+^, CD8^+^, CD4^+^CD25^high^, CD56^+^, CD19^+^, and CD69^+^ cells were isolated from peripheral blood mononuclear cells (PBMCs) from healthy blood donors by FACS sorting using a Becton Dickinson (BD) FACSAria cell sorting system and BD FACSDiva software v 4.1.2. Granulocytes and eosinophils were FACS sorted from whole blood following RBC lysis with ACK lysis buffer.

### microRNA microarray and data analysis

Total RNA from lesional skin of psoriasis patients (n = 3) and atopic eczema patients (n = 3) and skin of healthy individuals (n = 4) was isolated using TRIzol reagent (Invitrogen, Carlsbad, CA, USA) following the manufacturer's instructions. Two µg of total RNA from each sample were labeled using the miRCURY™ Hy3™/Hy5™ labelling kit and hybridized on the miRCURY™ LNA Array (v.8.0) (Exiqon, Vedbaek, Denmark). Signal intensities were normalized using the global Lowess regression algorithm. For subsequent analysis, we used the log2 of the background-subtracted, normalized median spot intensities of ratios from the two channels (Hy3/Hy5). To find consistently differentially expressed genes, the data were subjected to SAM analysis as described previously [9]. For visualization of differentially expressed miRNAs, a heat map was generated using TreeView (http://jtreeview.sourceforge.net). All microarray data reported in the manuscript is described in accordance with MIAME guidelines and have been deposited at EMBL-EBI (accession number: E-MEXP-1123).

### Quantitative real-time PCR

Total RNA of skin biopsies and cells was extracted using TRIzol reagent following the manufacturer's instructions. RNA from 20 different normal human organs was obtained from Ambion (FirstChoice® Human Total RNA Survey Panel). Quantification of miRNAs by TaqMan® Real-Time PCR was carried out as described by the manufacturer (Applied Biosystems, Foster City, CA). Briefly, 10 ng of template RNA was reverse transcribed using the TaqMan® MicroRNA Reverse Transcription Kit and miRNA-specific stem-loop primers (Applied Biosystems). 1.5 µl RT product was introduced into the 20 µl PCR reactions which were incubated in 384-well plates on the ABI 7900HT thermocycler (Applied Biosystems) at 95°C for 10 min, followed by 40 cycles of 95°C for 15 s and 60°C for 1 min. Target gene expression was normalized between different samples based on the values of U48 RNA expression.

### *In situ* hybridization

*In situ* transcriptional levels of miR-203 were determined on frozen sections (10 µm) of skin biopsy specimens from six psoriasis patients and six healthy individuals according to the manufacturer's instructions (Exiqon). Sections were hybridized o/n with digoxygenin-labeled miRCURY LNA probes (Exiqon) and incubated with anti-digoxygenin antibody conjugated with alkaline phosphatase for 1 h. Sections were visualized by using BM purple substrate together with 2 mM levamisole. The color reaction was performed o/n. We followed the protocol recommended by the manufacturer (Exiqon). The stained sections were reviewed with a Zeiss microscope.

### Immunohistochemistry

SOCS-3 protein expression was analyzed in skin from 9 psoriasis patients and 8 healthy control individuals. Cryostat sections (7 µm) from skin biopsies were stained with the ABC-ELITE (Vector Laboratories) immunohistochemical staining method, using rabbit anti-human SOCS-3 (Santa Cruz, USA), following the manufacturer's instructions.

### Western blotting

SOCS-3 protein was detected by immunoblotting with a mouse anti-human SOCS-3 antibody (Alexis Biochemical, Lausen, Switzerland). The protein levels were visualized by ECL (GE Healthcare) using horseradish peroxidase–conjugated Protein A/G (Pierce Chemical Co.)

### Statistical analysis

Parametric or nonparametric ANOVA (with Bonferroni's or Dunn's multiple comparisons test), and Student's t-test were used to determine the statistical significance of data.

## Supporting Information

Table S1. The evolutionary conserved targets of miR-203. Only those targets are shown that were predicted by three different algorithms independently (the intersection of miRanda, PicTar and TargetScan predictions).
(0.87 MB TIF)

Figure S1. Significantly enriched Gene Ontology. Putative targets of miR-203 were identified using TargetScan 3.0 algorithm. The occurrence of GO terms associated with miR-203 targets were analyzed using the Gene ontology Tree Machine and the Gene Set Analysis Toolkit (http://bioinfo.vanderbilt.edu/webgestalt; Vanderbilt University). Each GO category is represented by two bars. Significantly (p<0.01) enriched GO terms are labeled with red. The height of the green bar represents the gene number expected in the GO category based on the reference set selected (Expected number of genes in a specific GO category for an interesting gene set = total number of genes in the GO category for the reference set * Total number of genes in the interesting set/total number of genes in the reference set). The height of the red bar represents the number of genes observed in the GO category and also in the set of putative miR-203 targets.
(11.65 MB TIF)
