# Supplementary figures and images for: MicroRNAs: Novel Regulators Involved in the Pathogenesis of Psoriasis?
Source: PLoS One. 2007 Jul 11;2(7):e610. doi: 10.1371/journal.pone.0000610 (PMC1905940; doi:10.1371/journal.pone.0000610)

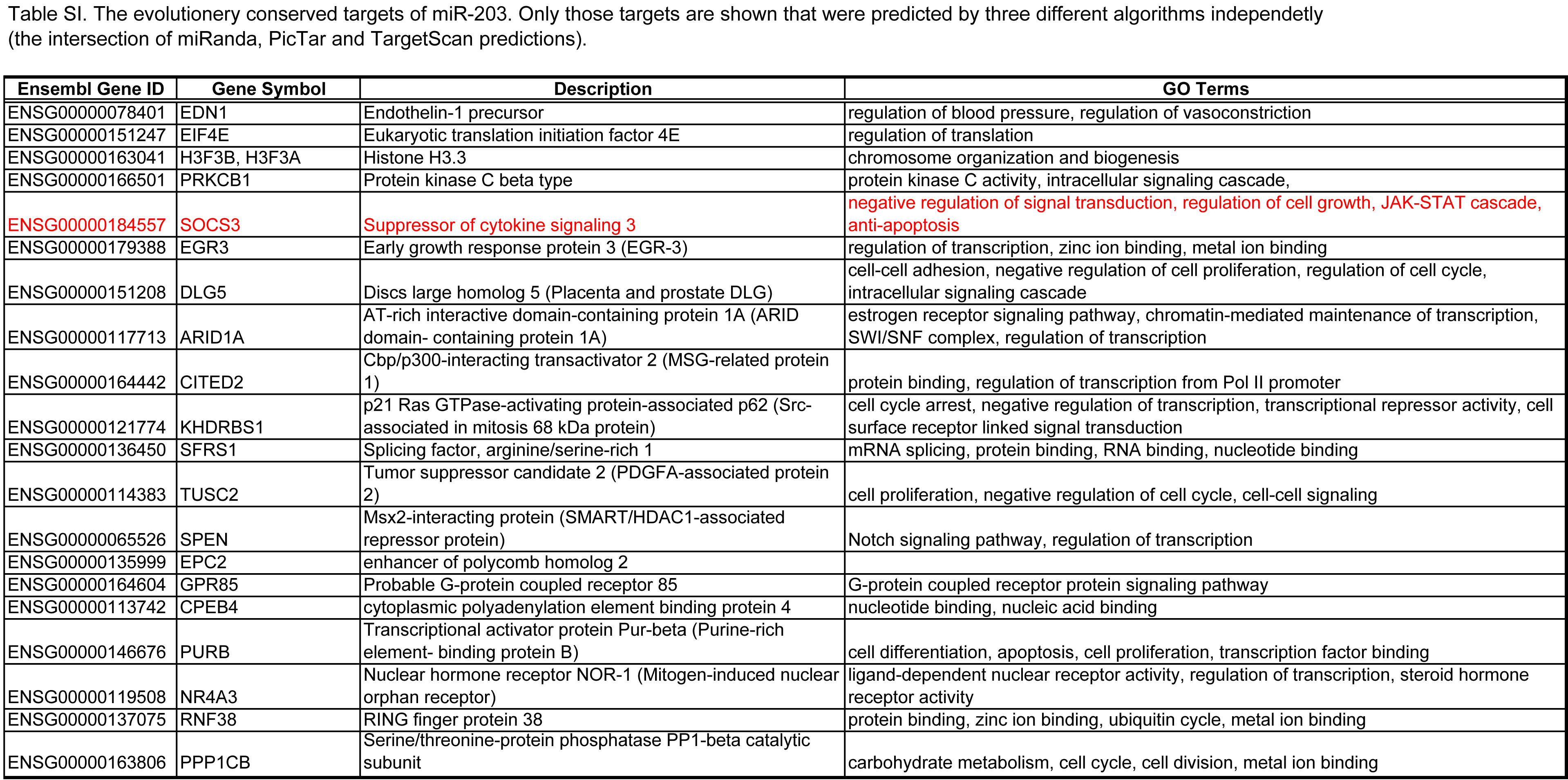

Supplement: Table S1. — The evolutionary conserved targets of miR-203. Only those targets are shown that were predicted by three different algorithms independently (the intersection of miRanda, PicTar and TargetScan predictions). (0.87 MB TIF) [file pone.0000610.s001.tif]

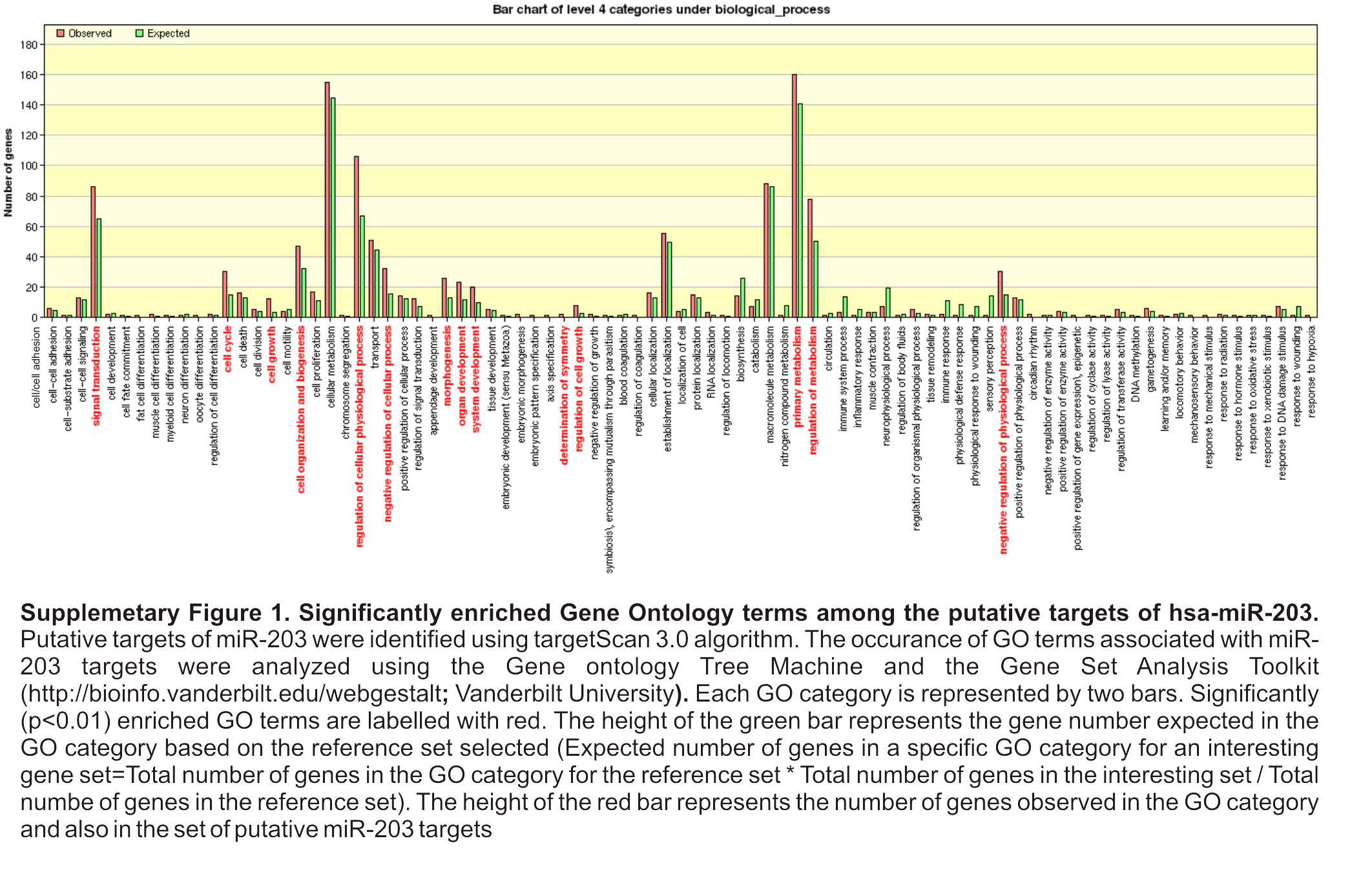

Supplement: Figure S1. — Significantly enriched Gene Ontology. Putative targets of miR-203 were identified using TargetScan 3.0 algorithm. The occurrence of GO terms associated with miR-203 targets were analyzed using the Gene ontology Tree Machine and the Gene Set Analysis Toolkit (http://bioinfo.vanderbilt.edu/webgestalt; Vanderbilt University). Each GO category is represented by two bars. Significantly (p<0.01) enriched GO terms are labeled with red. The height of the green bar represents the gene number expected in the GO category based on the reference set selected (Expected number of genes in a specific GO category for an interesting gene set = total number of genes in the GO category for the reference set * Total number of genes in the interesting set/total number of genes in the reference set). The height of the red bar represents the number of genes observed in the GO category and also in the set of putative miR-203 targets. (11.65 MB TIF) [file pone.0000610.s002.tif]
